# Methylenetetrahydrofolate Reductase Gene Variant (MTHFR C677T) and Migraine: A Case Control Study and Meta-analysis

**DOI:** 10.1186/1471-2377-11-66

**Published:** 2011-06-02

**Authors:** Zainab Samaan, Daria Gaysina, Sarah Cohen-Woods, Nick Craddock, Lisa Jones, Ania Korszun, Mike Owen, Andrew Mente, Peter McGuffin, Anne Farmer

**Affiliations:** 1 Department of Psychiatry and Behavioural Neurosciences, McMaster University, Hamilton, ON, Canada; 2 Medical Research Council (MRC) Social Genetic and Developmental Psychiatry Centre, Institute of Psychiatry, London, UK; 3 Department of Psychological Medicine, School of Medicine, Cardiff University, Cardiff, UK; 4 Division of Neuroscience, University of Birmingham, Birmingham, UK; 5 Barts and The London, Queen Mary's School of Medicine and Dentistry, London, UK; 6 Clinical Epidemiology and Biostatistics, McMaster University, Hamilton, ON, Canada

## Abstract

**Background:**

Migraine is a common disorder that often coexists with depression. While a functional polymorphism in methyleneterahydrofolate reductase gene (MTHFR C677T) has been implicated in depression; the evidence to support an association of MTHFR with migraine has been inconclusive. We aim to investigate the effect of this variant on propensity for migraine and to perform a systematic review and meta-analysis of studies of MTHFR and migraine to date.

**Methods:**

Individuals with migraine (n = 447) were selected from the Depression Case Control (DeCC) study to investigate the association between migraine and MTHFR C677T single nucleotide polymorphism (SNP) rs1801133 using an additive model compared to non-migraineurs adjusting for depression status. A meta-analysis was performed and included 15 studies of MTHFR and migraine.

**Results:**

MTHFR C677T polymorphism was associated with migraine with aura (MA) (OR 1.31, 95% CI 1.01-1.70, p = 0.039) that remained significant after adjusting for age, sex and depression status. A meta-analysis of 15 case-control studies showed that T allele homozygosity is significantly associated with MA (OR = 1.42; 95% CI, 1.10-1.82) and total migraine (OR = 1.37; 95% CI, 1.07-1.76), but not migraine without aura (OR = 1.16; 95% CI, 0.36-3.76). In studies of non-Caucasian population, the TT genotype was associated with total migraine (OR= 3.46; 95% CI, 1.22-9.82), whereas in studies of Caucasians this variant was associated with MA only (OR = 1.28; 95% CI, 1.002-1.63).

**Conclusions:**

MTHFR C677T is associated with MA in individuals selected for depression study. A meta-analysis of 15 studies supports this association and demonstrated effects across ethnic groups.

## Background

Migraine is a common primary headache disorder characterised by recurrent headaches associated with gastrointestinal, neurological and autonomic symptoms [1]. Migraine is sub classified further into several types, the most commonly noted are migraine with aura (MA) where aura symptoms, usually visual, precede the headache, and migraine without aura (MO). These common types of migraine are often reported to co occur with depression [2-5] making the screening for migraine in individuals with depression and vice versa an important clinical assessment with impact on diagnosis, treatment and prognosis. The screening for migraine in depression also has a significant impact for research where the findings may be confounded by the presence of another disorder. It is known that both conditions have a genetic background [6-8]. A common polymorphism from the MTHFR gene, the C677T, has been reported to be associated with both migraine and depression independently. The MTHFR gene is located on chromosome 1p36.3 and encodes for a key enzyme for the metabolism of folate and homocysteine [9]. The MTHFR 677C to T transmission is characterized by a point mutation resulting in a valine substitution for an alanine in this enzyme. Individuals with the homozygous (TT) state for this mutation showed higher levels of plasma homocysteine [9]. Homocysteine acts as an excitatory amino acid and may influence the threshold for migraine headache. High homocysteine levels are seen in individuals with migraine with aura [10].

A number of studies have found a significant association between the TT genotype and migraine [11-14]; especially MA [15] inconsistently [16,17].

The MTHFR C677T polymorphism has been reported to be associated with depression [18]; while other studies failed to show this association [19].

To our knowledge previous studies have investigated this gene variant in either cases of depression alone, or migraine alone without controlling for the presence of the other condition. We hypothesize that the MTHFR C677T polymorphism is a migraine gene and the association with depression is due to the presence of migraine in the individuals studied. In this study, we investigate the association between MTHFR C677T polymorphism and migraine in a sample of cases of depression and healthy controls. A meta-analysis of studies of this variant and migraine is also reported.

## Methods

### 1. Case Control Study

The study subjects were recruited for a genetic case-control association study of recurrent major depression, the Depression Case Control study (DeCC). The sample selection and recruitment have been described in detail [20]. Briefly, depression cases (18 years or older) of Caucasian origin with a recurrent depressive disorder were recruited from 3 sites in the UK (London, Cardiff and Birmingham). Control subjects were selected from the UK general practice based GENESiS study [21] and were included in the study if they were of Caucasian origin with no past or current psychiatric disorder. All subjects gave written informed consent. Local ethics committees of the study centres approved the study.

#### Depression Diagnosis

All depression cases were given a face to face diagnostic interview, the Schedule for Clinical Assessment in Neuropsychiatry (SCAN) [22], administered by trained interviewers to establish the diagnosis of recurrent depression. Control subjects were interviewed by telephone using the Past History Schedule [23] and were included if there was no evidence of past or present psychiatric disorder.

#### Migraine Diagnosis

For the lifetime diagnosis of migraine the structured migraine interview (SMI) [24] based on the ICHD [1] was used. Detailed questions about the presence of headache, severity, frequency, duration, site, type, aura symptoms and associated symptoms (nausea, vomiting, sensitivity to light and sound) were obtained. Depression cases were interviewed face to face and the control subjects were interviewed by telephone. Subjects were assigned a diagnosis of MA, MO, probable migraine (PM), no recurrent headaches and non-migraine headaches.

#### DNA Samples

DNA samples were obtained from whole blood or buccal epithelial cells for molecular genetic analysis. Whole blood samples were collected at the time of the interview for all cases with depression that consented for the genetic portion of the study. Interviewers collected 25 ml of blood from peripheral veins in the anticubital fossa. Blood samples were collected in Monovettes tubes containing EDTA. Each tube was rolled gently after blood collection was completed and stored at room temperature until the end of the interview where it was then transferred to the local lab for storage at -20°C freezer in upright position. In addition, blood drops from Monovettes tubes were placed on Buffy Coat. All samples (tubes and Buffy Coat) were labelled with a unique ID number and bar codes were placed on the tubes and Buffy Coat. Control subjects were sent cheek swabs (labelled with unique barcode ID) and instructions on how to obtain buccal swab samples and were supplied with return addressed envelopes. DNA was extracted in house from both whole blood and buccal mucosa samples following validated procedures as described earlier [19]. Genotyping of the MTHFR C677T SNP rs1801133 was performed by KBioscience (Hoddesdon, Hertz, UK, http://www.kbioscience.co.uk), blinded to the phenotype status. Full details of the genotyping methods and primers design were previously described [19].

#### Statistical Analysis

Data were entered into SPSS version 14 for Windows and statistical analyses were performed using chi square statistic and logistic regression analysis. Genotype and allele frequencies were investigated for association with migraine using contingency tables and chi square statistic. To test for specific association between MA and MTHFR C677T, logistic regression was used with MA as dependent variable and age, sex and depression were entered as co variables.

### 2. Meta-analysis

Two investigators (ZS and AM) searched databases (Pubmed, Google Scholar and Embase) for studies of MTHFR and migraine up to July 2010 (published in English). Search terms included "MTHFR", "methylenetetrahydrofolate reductase", "gene", "genetic variant", "polymorphism", "rs1801133", "headache", "migraine", "migraine with aura" and "migraine without aura". Studies were included if they employed a case-control design, migraine as the primary outcome and data were available to extract or calculate OR and 95% CI. Data from primary studies were extracted by ZS and AM independently and entered into excel database. Any discrepancy between investigators' findings was clarified and agreement reached by consensus. Fifteen studies including the current case control study were included in the analysis. These studies are presented in Table 1. For the statistical analysis, we used commercially available statistical software (Comprehensive Meta Analysis software, version 2.2 [Biostat, Englewood, New Jersey]).

**Table 1:** Migraine and MTHFR case control studies

Migraine	Population	Cases	Controls	OR (95%CI)	Reference
MA	British Caucasian	124	1725	1.3 (1.01-1.70)	Current

MA	North Indian	67	150	1.295^"a" ^(0.676-2.478)	[44]

MA	Portuguese	78	397	0.48 (0.18-1.23)	[29]

MA	Italian	100	105	2.48 (1.11-5.58)	[39]

MA, MO	Italian	105 (90 MO and 15 MA)	97	MA: 2.16 (0.64-7.41) MO: 2.04 (0.98-4.22)	[45]

MA	Italian	33 (5-17 yr old)	66 (Adults)	2.18 (0.68, 7)	[46]

MA ^"b"^	USA Caucasian female only	1275	23726	0.79 (0.65-0.96)	[26]

MA	Japanese	22	261	6.5 (2.5-16.8)	[12]

MA, MO	Turkish	93(23 MA; 70 MO)	136	MA: 3.05 (0.39-24.51) MO: 7.44 (1.70-32.34)	[11]

MA, MO	Spanish Caucasian	230 (78 MA and 152 MO)	204	MA: 1.37 (0.63-2.94) MO: 0.59 (0.27-1.24)	[15]

MA	Australian Caucasian	168	269	2.54 (1.37-4.71)	[13]

MA, MO	Spanish Caucasian	329 (138 MA and 191 MO)	237	MA: 2.4 (0.8-7.2) MO: 0.28 (0.1-0.8)	[37]

MA	Dutch	187	1212	2.05 (1.2-3.4)	[14]

MA	German	656	625	0.87 (0.61-1.25)	[16]

MA	Finnish	898	900	0.98 (0.84-1.15) ^"c"^	[17]

MA migraine with aura, MO migraine without aura. OR for T/T genotype of MTHFR C677T polymorphism. ^"a" ^CT genotype is the reference point. MA n = 67, 0% TT genotype, 69.9% CC, 30.1% CT. healthy controls n= 150, TT 2%, CC 72% and CT 26%.

^"b" ^self reported migraine with aura. ^"c" ^OR for T allele frequency in cases and controls. 12.7% of cases did not fulfil criteria for MA based on the International Headache Society Criteria for MA. MA here is therefore used as a broad definition.

Statistical heterogeneity across studies was assessed using the Q statistic, with significant heterogeneity for all of the migraine outcomes. Summary estimates were calculated using a general variance-based method (random-effects model) with 95% CI [25]. Stratified analyses were also conducted by ethnic ancestry (European or non-European). Since one study included women participants only, a sensitivity analysis was conducted whereby this one study was excluded from the analysis to determine whether the parameter estimates are altered.

## Results

The sample characteristics are presented in Table 2. Both the migraine and non-migraine groups were in their 5^th ^decade of life with mean age of 47 years. Women were more likely to have migraine and individuals with migraine were more likely to be depressed.

**Table 2:** Sample Characteristics

Categorical Variables	No Migraine N = 1402	Any Migraine N = 447	**χ**^**2**^	p
Female N [%]	951 [59.7]	284 [76.3]	60.3	< 0.0001

Depression N [%]	851 [53.5]	286 [76.9]	105.4	< 0.0001

Married/cohabiting N [%]	422 [26.5]	121 [32.5]	2.3	0.13

≥ 1 Child N [%]	479 [30.1]	65 [17.5]	4.4	0.04

Currently employed N [%]	263 [16.5]	55 [14.8]	1.9	0.16

≥ 12 years education N [%]	207 [13.0]	77 [20.7]	0.36	0.55

**Continuous Variables**			**t**	**p**

Age in year [SD]	47.7 [± 11.14]	46.9 [± 11.7]	1.4	0.16

SD Standard Deviation

Genotype and allele frequencies for the MTHFR gene C677T SNP were analysed for 810 (95% genotyping reaction success) psychiatrically healthy controls and 1039 (83% genotyping success rate) depression cases. The controls' genotype frequencies were in Hardy-Weinberg Equilibrium (HWE) (χ^2 ^= 0.01, df = 1, p = 0.92).

### MTHFR C677T polymorphism and migraine

Allele and genotype frequencies for the MTHFR C677T variant were investigated in migraine. Migraine categories were assigned as follows:

• No migraine headache including no recurrent headache and non-migraine headaches.

• Migraine including MA, MO and PM.

• MA only.

Table 3 shows the allele and genotype frequencies for the different migraine categories. T allele and TT genotype were more frequent in MA. Significant statistical difference was seen in T allele frequencies between MA and no migraine groups (χ^2 ^= 5.45, df = 1, p = 0.02, OR = 1.37, 95% CI 1.04 - 1.80). No statistical difference was detected between migraine (including MA, MO and PM) and no migraine in the T allele frequency (36.7% vs 34.2%, χ^2 ^= 1.9, df = 1, p = 0.17, OR = 1.12, 95% CI 0.95 - 1.31). However, TT genotype was significantly different between MA and no migraine groups (χ^2 ^= 4.87, df = 1, p = 0.027, OR = 1.8, 95% CI 1.03 - 3.16).

**Table 3:** rs1801133 genotype and allele frequency

Genotype	No Migraine N [%]	Any Migraine N [%]	Migraine with Aura N [%]
TT	181 [12.9]	62 [14.0]	23 [18.5]

CT	596 [42.5]	204 [46.0]	57 [46.0]

CC	625 [44.6]	181 [40.0]	44 [35.5]

Alleles			

T	958 [34.2]	328 [36.7]	103 [41.5]

C	1846 [65.8]	566 [63.3]	145 [58.5]

The effect of sex was explored in the analysis and showed excess T allele frequency in women with MA (χ^2 ^= 8.22, df = 1, p = 0.004, OR = 1.53, 95% CI 1.13 - 2.07) and TT genotype (χ^2 ^= 7.47, df = 1, p = 0.006, OR = 2.20, 95% CI 1.19 - 4.07).

In order to investigate the association between MA and MTHFR C677T adjusting for sex and depression status we performed logistic regression analysis using an additive model with MA as the dependant variable and age, sex and depression as the explanatory variables. Excess T allele was significantly associated with MA (OR 1.31, 95% CI 1.01-1.70, p = 0.039). Summary of the logistic regression results is shown in Table 4.

**Table 4:** Summary results of logistic regression analysis.

Variable	OR	95% CI	p
Age (year)	1.0	0.98-1.02	0.96

Sex	2.7	1.64-4.40	< 0.0001

Depression	3.7	2.29-6.13	< 0.0001

rs1801133	1.3	1.01-1.70	0.039

Presence or absence of migraine with aura is the dependent variable

OR Odds Ratio

### Meta-analysis

All studies (15 [100%]) (Table 1) assessed MA, while some (4 [26.7%]) also evaluated MO, and 1 (6.7%) examined tension headache. In total, the 15 studies included N = 4,374 migraine cases and N = 30,110 controls. Table 1 summarizes the characteristics and results of the included case-control studies by migraine type. Among the 15 studies included in the meta-analysis, 12 studies enrolled participants of European ancestry, while 3 studies recruited participants of non-European ancestry. Almost all of the studies (14 [93.3%]) included both men and women.

**Figure 1:**
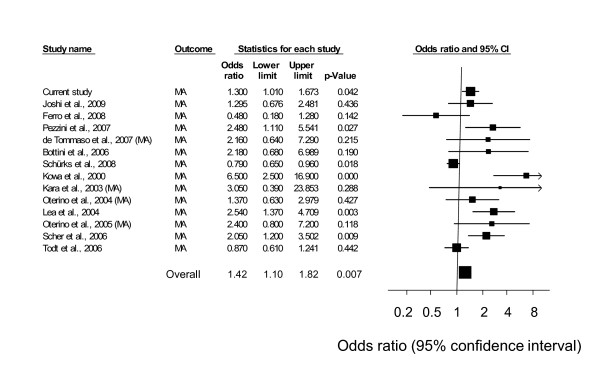
**Meta-analysis results of studies examining the effect of the C667T polymorphism on MA headache**.

**Figure 2:**
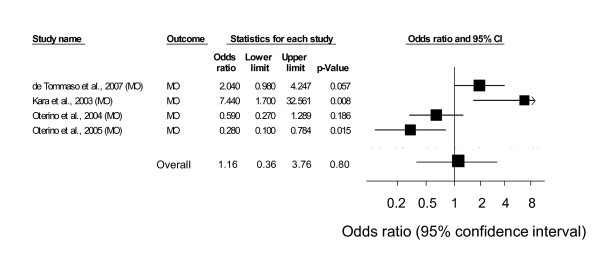
**Meta-analysis results of studies examining the effect of the C667T polymorphism on MO headache**.

The pooled analyses of case-control studies employing an additive model showed that T allele homozygosity was significantly associated with MA (OR = 1.42; 95% CI, 1.10-1.82) and total migraine (OR = 1.37; 95% CI, 1.07-1.76), but not with MO (OR = 1.16; 95% CI, 0.36-3.76) (Table 5). In a sensitivity analysis that excluded 1 study, which enrolled only women, the parameter estimates remained virtually the same (Table 5). Forest plots summarizing the effect of this variant on migraine in each of these studies are shown in Figure 1 for migraine with aura and Figure 2 for migraine without aura. Significant heterogeneity across studies was found both overall (p < 0.0001) and by migraine type (p < 0.0001 for each type).

**Table 5:** Meta-analysis summary odds ratios (95% CI) for the association of the T allele homozygosity with migraine

	All migraine	MA only	MO only
	(15 studies)	(15 studies)	(4 studies)

	N = 4,365 cases	N = 3,862 cases	N = 503 cases
	N = 30,110 controls	N = 30,110 controls	N = 674 controls

All studies	1.37 (1.07-1.76) ^"a"^	1.42 (1.10-1.82) ^"a"^	1.16 (0.36-3.76)

Excluding 1 study of women only ^"b"^	1.46 (1.11-1.92) ^"a"^	1.54 (1.17-2.02) ^"a"^	1.16 (0.36-3.76)

Ethnic ancestry			

European	1.20 (0.95-1.53)	1.28 (1.002-1.63) ^"a"^	0.73 (0.24-2.24)

Non-European	3.46 (1.22-9.82) ^"a"^	2.81 (0.84-9.44)	------- ^"c"^

^"a" ^Statistically significant.

^"b" ^All studies included men and women except for Schurks et al 2008 which enrolled only women.

^"c" ^No summary OR was computed, since there was only 1 study.

Table 5 shows the summary estimates for each migraine type by ethnic origin. In studies of European ancestry, there was a significant association between TT genotype and MA (OR = 1.28; 95% CI, 1.002-1.63), but not with MO (OR = 0.73; 95% CI, 0.24-2.24) or total migraine (OR= 1.20; 95% CI, 0.95-1.53). Conversely, in studies of non-European origin, the TT genotype was not significantly associated with MA (OR = 2.81; 95% CI, 0.84-9.44) but was associated with total migraine (OR= 3.46; 95% CI, 1.22-9.82), and there was only 1 study that evaluated MO.

**Figure 3:**
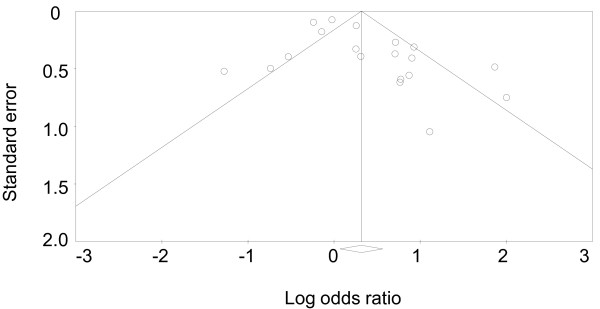
**Funnel plot of case-control studies of the C667T polymorphism and headache**.

An assessment of the funnel plot of case-control studies of the C667T polymorphism and migraine suggested a slight publication bias (Figure 3). Point estimates from smaller studies, with greater variability, were more likely to be distributed on the positive side of the mean effect size. Egger's regression test showed borderline evidence of publication bias (Egger test of the intercept, P = 0.03).

## Discussion

We report a significant association between a common SNP (rs1801133) in the MTHFR gene and MA in a well-characterized sample of depressed cases compared to psychiatrically healthy controls. Previous reports have shown an association between this SNP and depression. In fact, the association between MTHFR polymorphism and MA remained significant after adjusting for depression suggesting that this association is unique to MA. No statistically significant association was seen between this SNP and depression or MO in this study. The association between MTHFR C677T polymorphism and MA was stronger in women, however remained significantly associated with MA after controlling for sex, age and depression. Several studies suggested an association between MTHFR C677T polymorphism and migraine as described earlier. Similarly, our meta-analysis of 15 case-control studies showed that the T allele is significantly associated with MA and total migraine, the association with total migraine is most likely accounted for by the presence of migraine with aura. Conversely other studies found the TT genotype protective for MA [26-29]. A meta analysis [30] pooled data from 2961 cases of migraine reported a significant association between TT genotype and MA only (OR 1.30, 95% CI 1.06-1.58). More recently, a meta-analysis of studies published up to March 2009, reported a significant association between migraine with aura and MTHFR C677T polymorphism (OR 1.48, 95% CI 1.02-2.13) that was mainly driven by non-Caucasian populations [31]. Our meta-analysis included 2 additional studies and found that this genetic variant is associated with total migraine in non-Caucasian populations and with migraine with aura in Caucasians. Furthermore, it has been suggested that this polymorphism was also associated with depression [18,32-35], we have reported previously that there was no significant association between depression and this SNP [19] and we argue that the previous reports of MTHFR C677T association with depression can be explained by the high prevalence of migraine in depressed subjects included in these studies. None of the studies reported have screened subjects for migraine. Since migraine can be present in almost a third of participants [2], it is likely that the association between MTHFR C677T and depression is actually an association with migraine.

In this depression case control study, we found migraine to be significantly associated with excess T allele and TT genotype of the MTHFR C677T polymorphism. This association was specific only to MA. Broad migraine (including MA, MO and PM) was not significantly different than non-migraine headaches in their T allele and TT genotype distribution. This trend was also shown in previous studies in different populations as described earlier. The results were not always consistent however and other studies reported negative findings. A relatively large study failed to show an association between migraine and MTHFR C677T polymorphism [17]. Such contradictions can be explained in part by methods of identification of migraine, the different populations (clinical, epidemiological samples), age and sex of the study participants, ethnic variations and geographic sites included in these studies. Studies have shown that there are genetic variations across different populations and geographic locations; these variations may have a significant role in genetic studies findings when different populations were included. A small Japanese study (n = 22) was the first to report a significant association between MTHFR C677T and MA [12] replicated by several other small studies (Korean n = 34 [36], Spanish n = 60 [37] and Italian n = 33 [38]) while large German (n = 656) and Finish (n = 898) studies failed to show significant association between MA and this polymorphism [16,17]. These observations were in parallel with our meta-analysis by subgroups, which showed a strong and significant association between TT genotype and migraine in non-Europeans, although we also observed a modest but significant association with MA among Caucasians. This ethnic specific association between a genetic variant and phenotype highlights the importance of ethnic variations in genetic studies and the need to expand studies beyond one ethnic group. In addition, ethnic variation may be a reflection of health behaviour and life style factors such as dietary habits and policies on folic acid fortification of diet and supplements. In addition to the different population studied, there is also a significant variation in migraine case identification that added further heterogeneity to the reported studies. The diagnosis of migraine, like depression, is mainly based on careful history taking of specific symptoms that show repeated patterns over time and lack of significant findings in physical examination. Objective measures to confirm or deny the diagnosis of migraine are not yet available and therefore the methods of identification of migraine cases are important sources of heterogeneity and conflicting results in the literature of migraine genetics. Our findings of significant association between MA and MTHFR C677T in a well characterised study of 1849 subjects (migraine cases = 447) replicates earlier studies of moderate size [13,14,39] suggesting a role for the MTHFR gene in migraine especially MA. Further meta analysis of 13 studies reported a pooled OR = 1.48, 95% CI 1.02-2.13, for MA only [31]. This may also indicate that the MTHFR gene effect is associated with aura symptoms specifically rather than migraine in general. It will be interesting to investigate homocysteine (a risk factor for stroke [40]) and folate levels, low folate levels have been reported in depression [41], in subjects with migraine with aura to confirm the association between MTHFR mutation and its function by measuring the end products of the enzyme. A small open label trial of folic acid administration in children with migraine, hyperhomocysteinaemia and MTHFR C677T polymorphism (TT genotype) reported a reduction in migraine symptoms in this population [42]. In a study of 136 individuals with migraine with aura compared to controls, homocysteine level was higher in the migraine group [10]. The homocysteine level was also higher in MA compared to MO [43]. Randomized controlled trials are needed to confirm such findings, the results of which will be valuable for clinical practice in the management of migraine and depression.

Our results showed that excess T allele and homozygous TT genotype of the MTHFR gene C677T polymorphism is associated with increased risk for MA regardless of depression status. Our case control study showed that individuals carrying this genetic variant are 30% more likely to have migraine with aura and meta-analysis strengthened this observation and reported 42% increase in risk for such individuals, however it is important to keep in context that this 42% risk increase for MA is only in individuals carrying this genetic variant, the number of which is relatively small compared to the general population. The importance of our findings can be focused into 2 main areas:

1^st ^studies of the MTHFR C677T variant are common especially in psychiatric and cardiovascular fields. These studies, we encourage, must take into consideration the common co occurrence of migraine in these disorders and the strong and reproducible association between this variant and migraine with aura. It is advisable therefore to screen and adjust for migraine in these studies to avoid spurious associations.

2^nd ^individuals carrying this variant (although a small number of individuals are carrying the TT genotype variant) are at 42% increased risk for migraine with aura. In the light of increasing interests in personalised medicine, adding folate supplements to individuals at risk (a specifically targeted intervention for population at risk) may mitigate future development of migraine with aura.

The main strengths of our study are the use of validated diagnostic tool to identify migraine and the use of direct interviewing of all participants. Our study participants are also relatively homogenous (of Caucasian ethnicity, residing in the UK) thus reducing population stratification that can have a confounding effect. There are however certain weaknesses to our study. The study recruitment was designed to select depressed cases, which may have led to ascertainment bias. However migraine was systematically screened for in all participants and we have adjusted for depression status in our analysis. In addition our sample size did not allow for subgroup analysis of each headache type and therefore we are not able to report whether there is any association between this SNP and other types of primary headache disorders in addition to MA such as tension type headache.

## Conclusions

The current study adds to the growing evidence for the role MTHFR C677T gene variant in migraine with aura. Our meta-analysis showed a significant association between this genetic variant and MA in Caucasian population and overall migraine in non-Caucasian population, although the effect in this case is very likely to be due to MA than migraine in general. Heterogeneity of studies remains a significant cause of conflicting results and ethnic variations in genotype-phenotype association must be examined closely.

## Abbreviations

MA: migraine with aura; MO: migraine without aura; MTHFR: methylene tetrahydrofolate reductase; SNP: single nucleotide polymorphism

## Competing interests

The authors declare that they have no competing interests.

## Authors' contributions

ZS conceived the migraine study, designed the migraine interviews, collected data, analyzed data and wrote the 1^st ^draft. With AM. ZS also performed literature search for meta-analysis. DG and SCW performed all DNA related lab experiments. NC, LJ, AK and MO are investigators on the depression case control study, designed the original study, were responsible for the study conduct and recruitment, supervision of study personnel and contributed to the manuscript. AM performed literature search for the mea-analysis and run the analysis for this part of the study, he also contributed to the manuscript writing. PM and AF are the depression case control study PIs and the supervisors of the 1^st ^author; they guided the study with significant input into the design of the migraine study and selection of gene variant, they also contributed to the writing of the manuscript and provided the resources for the migraine study. All authors read and approved the final manuscript.

## Pre-publication history

The pre-publication history for this paper can be accessed here:

http://www.biomedcentral.com/1471-2377/11/66/prepub
